# Authorship Weightage Algorithm for Academic Publications: A New Calculation and ACES Webserver for Determining Expertise

**DOI:** 10.3390/mps4020041

**Published:** 2021-06-09

**Authors:** Wei-Ling Wu, Owen Tan, Kwok-Fong Chan, Nicole Bernadette Ong, David Gunasegaran, Samuel Ken-En Gan

**Affiliations:** Antibody & Product Development Lab, EDDC-BII, A*STAR, Singapore 138672, Singapore; Wu_Weiling@eddc.a-star.edu.sg (W.-L.W.); ozruen@gmail.com (O.T.); kwokfong29@gmail.com (K.-F.C.); 1701734D@student.tp.edu.sg (N.B.O.); Davidsegar2@gmail.com (D.G.)

**Keywords:** authorship, natural language processing, score, weightage

## Abstract

Despite the public availability, finding experts in any field when relying on academic publications can be challenging, especially with the use of jargons. Even after overcoming these issues, the discernment of expertise by authorship positions is often also absent in the many publication-based search platforms. Given that it is common in many academic fields for the research group lead or lab head to take the position of the last author, some of the existing authorship scoring systems that assign a decreasing weightage from the first author would not reflect the last author correctly. To address these problems, we incorporated natural language processing (Common Crawl using fastText) to retrieve related keywords when using jargons as well as a modified authorship positional scoring that allows the assignment of greater weightage to the last author. The resulting output is a ranked scoring system of researchers upon every search that we implemented as a webserver for internal use called the APD lab Capability & Expertise Search (ACES).

## 1. Introduction

The surge of research article publications in recent years and the use of jargon often make it challenging for those not within the field to navigate through them to find and discern experts. Yet, finding *bona fide* experts continues to grow in importance in a world of ever-increasing misinformation and fake news that can lead to serious consequences [1], made evident during the COVID-19 pandemic [2]. This need to discern the right experts in the many public/private sectors for consultancy and collaborations can also make the difference between success and failure. However, the definition of ‘expert’ is highly contentious, varying from experience (time in the field) to the very nuanced measurement of achievements that can include social impact, journal impact factors, citations, patents, and commercialized products, among others, that have different values to the various needs. Addressing academic usage alone, this work will focus on research publications given their public availability for filtering of expertise. Although publicly available, navigating through publications in no simple task, especially when considering the authorship issues discussed in previous articles [3,4,5,6].

Currently, there is a plethora of online platforms that help identify experts based on research publications that include ResearchGate, Google Scholar, Web of Science, ORCID, and Publons, among others. To an extent, this also includes LinkedIn, which, although not publication-based, targets a broad user base beyond academia. Although expert identification based on publications are convenient, caveats exist in that the contribution of co-authors are typically assumed to be equal in these scoring/ranking platforms [7]. A contentious topic that is still debated in academia [7,8], authorships are originally intended to reflect the level of contribution by the authors to the research [8,9,10,11,12], and would therefore be a good filter in determining expertise. In fact, authorship is one of the key issues discussed in collaborations, with dedicated resources providing advice to younger scientists [13].

Many scoring and ranking methods, such as those in the existing platforms, lack features to differentiate highly collaborative academics with only middle authorships from high performing academics with a similar number of publications, but as mostly first or last authors. Although discerning eyes could quickly differentiate that the latter is more likely to have higher expertise than the former, this discernment is not reflected in the current metrics nor conveniently available in the current platforms.

To include authorship as an added discernment filter, we incorporated a new scoring method that can credit the last author as much as the first, and implemented it as a web server for assessment. Incorporating a Common Crawl using fastText methods to group search input keywords, the problem of jargon usage is mitigated through related word search. The resulting webserver complements our other digitalization efforts [14] in building an expert identification system to be used by journals and grant offices and to identify suitable peer reviewers. As a demonstration, we extracted the 2018 Google Scholar data of ~700 researchers from the Agency for Science, Technology, and Research, Singapore, and incorporated them into the APD lab Capability and Expertise Search (ACES) webserver.

## 2. Materials & Method

### 2.1. Scoring Method

The default scoring method for publication co-authors ‘N’ uses the harmonic authorship credit model previously described by Hagen [15] that gives the highest weightage to the first author, followed by the subsequent authors. The position of the author of interest (p) is determined from the left of the author list and is an element of positive real numbers (1, 2, 3, 4, …).

(1) $$\mathrm{score}=\frac{\frac{1}{p}}{\underset{i=1}{\overset{N}{\Sigma }}\frac{1}{i}}$$

With the selection of the option to credit the last author and second to last with equal weightage as that of the first and second, respectively, the author score would be calculated using Equations (2)–(5), represented by the position of the author of interest (p) in the (i) first or last position:

(2) $$\mathrm{score}=\frac{1+\frac{1}{2}}{2\left(\underset{i=1}{\overset{N}{\Sigma }}\frac{1}{i}\right)}$$

(ii) second or second last position:

(3) $$\;\mathrm{score}=\frac{1+\frac{1}{2}+\frac{1}{3}}{6\left(\underset{i=1}{\overset{N}{\Sigma }}\frac{1}{i}\right)}$$

(iii) any position between the second and second last position of the author list:

(4) $$\mathrm{score}=\frac{\frac{1}{p+2}}{\underset{i=1}{\overset{N}{\Sigma }}\frac{1}{i}}$$

and an exception for (iv) N = 3, where the author of interest is in the middle position.

(5) $$\mathrm{score}=\frac{\frac{1}{3}}{\underset{i=1}{\overset{N}{\Sigma }}\frac{1}{i}}$$

### 2.2. WebServer Design

ACES was developed using hypertext markup language (HTML), cascading style sheets (CSS), and JavaScript for the front-end and Python version 3.7.5 using Flask version 1.1.1 framework for the back-end server.

When the user first types in a subject keyword on the main page, ACES retrieves publications in the database by relevance and assigns a score to the publication based on the distance to the keyword, followed by tabulating the scores of the researcher based on the authorship position. The results are then displayed showing the ranked scores of the relevant researchers. The total number of publications, citations, and most related publications pertaining to the search query are then displayed under each author.

### 2.3. Database

The sampling of 700 public Google Scholar profiles of researchers affiliated with the Agency for Science, Technology, and Research (A*STAR), Singapore, were collected in 2019 and stored in a NoSQL database. The back-end server receives the data in the JavaScript Object Notation (JSON) format containing names, affiliations, total number of citations and publications, research article titles with the authors list, and 30 most important keywords associated with the researcher. Applying the term frequency–inverse document frequency (tf-idf), a numerical statistic to determine the importance of a word in a given document [16] onto the publication titles as a whole, the relevance to keywords could be computed.

### 2.4. Search

The search algorithm consists of three main phases: query processing, researcher retrieval, and ranking. It utilizes the Natural Language Toolkit (NLTK) version 3.4.4 and Gensim version 3.8.0 libraries.

#### 2.4.1. Query Processing

**Figure 1:**
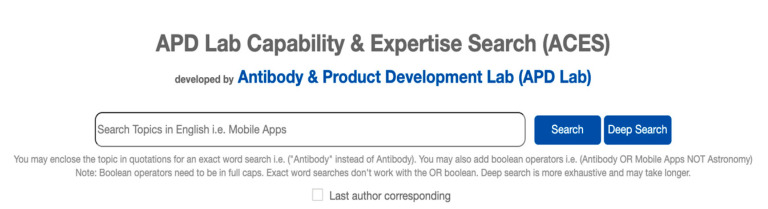
Main page of the website with the option to check the last author corresponding option.

Upon the search (see Figure 1), the search query processing phase starts by filtering out punctuation marks and stop words i.e., insignificant words that appear frequently in English for framing sentences [16]. Precompiled stop words were supplied by NLTK and further modified to enable stringent keyword filtering and increase the accuracy of the search query.

#### 2.4.2. Retrieval of Researchers

ACES scores the relevance of the processed search query in the keywords of every researcher. Using Gensim NLP library (https://radimrehurek.com/gensim/ (last accessed on 28 April 2021)), two-million-word vectors with 300 dimensions were loaded on the back-end server. These word vectors were trained on Common Crawl (https://commoncrawl.org (last accessed on 28 April 2021)) using fastText [17], and are used to map the processed query to its corresponding values.

By computing the cosine similarity between word vectors of the search query and the 30 most important keywords of each researcher, a relevancy score is calculated. For a faster result output, a batch of the top 20 high-scoring researchers based on keywords are first shown.

#### 2.4.3. Ranking

The ranking phase comprises three steps: word processing, article scoring, and the ordering of researchers. The publication titles are processed in the same way as the search query where the punctuation marks and stop words are filtered out before the cosine similarity between the word vectors are computed. This is followed by the authorship computation which utilizes the harmonic allocation method [15].

**Figure 2:**
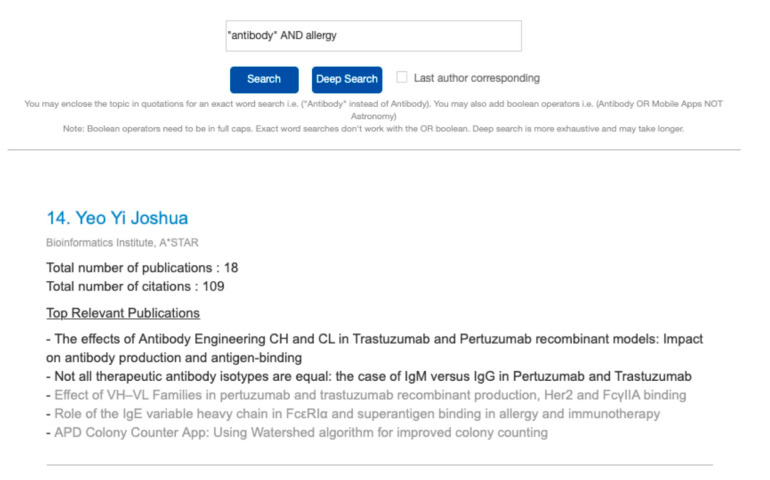
Example of a researcher in the results page.

Excluding publications with low relevance to the query keyword, overall scores are assigned to the researcher based on the sum of relevance and authorship position scores. The top 20 researchers are ranked based on the combined scores and displayed on the webpage together with their top five relevant publications and other information available in the database. Publications of low relevance are greyed out, allowing the user to focus on only computationally relevant ones (see Figure 2). The researcher names and publications are shown as hyperlinks to a Google search for easily follow-up with only twenty researchers displayed per page.

## 3. Results and Discussion

In this work, we built the ACES web server as a resource for discerning and finding academic experts that takes into account the authorship positions. While there are existing algorithms (such as the harmonic authorship credit model), the last author is insufficiently credited. This is especially so in the many academic disciplines where the research group lead or Ph.D. supervisor who conceptualized and directed the research, is the last corresponding author. In some cases of collaborations between groups, the collaborating research group senior author occupies the second to last position. For this reason, we allowed the assigning of weightage equivalent for the second last author equivalent to that of the second author for ease of implementation even though there are many situations where the second to last author may deserve as much weightage as that of the last author. We also acknowledge that there are also many cases where the second to last author may not necessarily be a senior author, but given that the option should be applied to senior scientists, usage discretion can minimize such mis-attribution. 

### 3.1. Exact Word Matching and Autocorrect

In addressing jargon usage in publication titles, a natural language processing toolkit for word matching of the search keyword was added alongside other utility features. The search algorithm in ACES excludes phrases or words with ambiguous meanings to prevent confounding the scoring. In cases where the search of the jargon word is desired, the user can use exact word searches with flanking double quotation marks.

**Figure 3:**
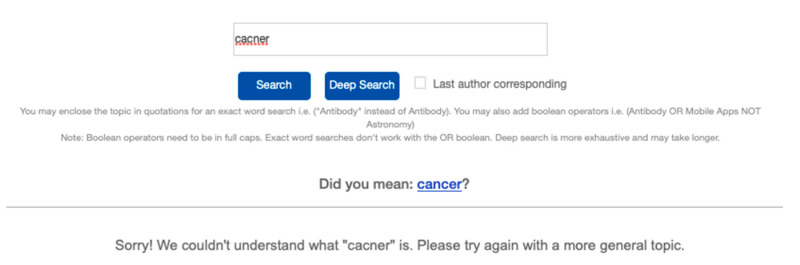
Autocorrect suggestion for the misspelled word cancer.

Since typographical/spelling errors are inevitable, we incorporated a suggested word feature using the Python package autocorrect version 0.4.4. Although a search with the error would still be performed, an autocorrect suggestion below the search bar will be displayed with the results (see Figure 3) to which a new search with the suggested correct word can be re-performed by clicking on the hyperlink.

### 3.2. Deep Search

ACES was designed to provide nearly instantaneous results with a slight compromise of accuracy. For higher accuracy, a deep search feature was could be performed (see Figure 1 and Figure 3) to skip the phase of fetching just the top 20 researchers based on their keywords, but to perform matching throughout the whole database. While this will take a slightly longer time to compute the results, all of the researchers in the database would be screened. We have incorporated this default fast search to facilitate future growth with researcher data many folds more than the current small database of about 700 researchers, which may result in long processing times.

### 3.3. Authorship Contribution

The reflection of contribution by authorship positions varies in different research fields [11,12], requiring tweaks to the harmonic allocation [15] method for many academic fields where the last author position is the principal investigator or research lead who can be just as important, if not more important, than the first author [12]. To allow for better credit to the last authors, a checkbox below the search bar labelled “Last author corresponding” (see Figure 1) was included as an option. This allows the weightage given to the last author to equal that of the first author and the second to last with that of the second. This option will yield different ranking results for researchers with more second to last or last author publications. While it remains contentious whether the last authors should have more or equal credit to the first authors, and perhaps more so for the second to last author, we have attributed the weightage as such for ease of implementation. Nonetheless, this option should be used only for relevant fields to prevent confounding the outcomes. If the search intends to search of senior authors, enabling the option in the relevant field will allow better attribution, even in situations where they appear as second to last authors in some publications. 

**Figure 4:**
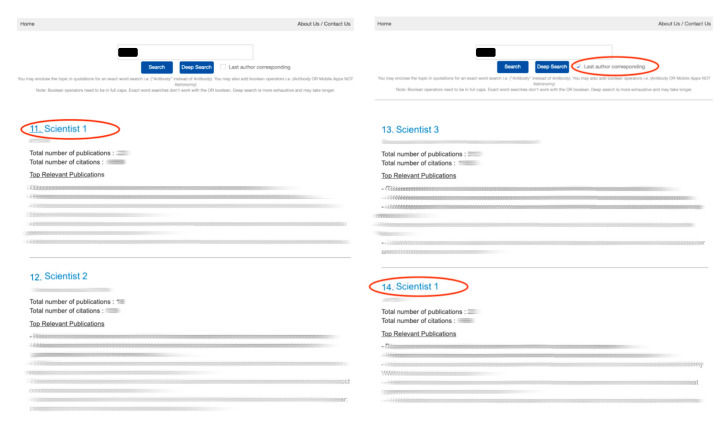
Search result display of “allergy” before (**left**) and after (**right**) the last author option is selected, showing different ranking when considering last authorship.

Since the general user is likely to focus only on the top few ranked individuals for expertise, the order can matter. As a blinded demonstration (Figure 4), when applying the last author weightage, there would be a reordering of Scientist 1 who has more middle author publications than the other preceding researchers. While the movement here is perhaps small, such reshuffling could be more pronounced in a larger list, demonstrating the relevance of this feature as an added discernment filter.

We acknowledge that the scoring method is not comprehensive and that there remain many variations in last authorships and the possibility of slighting the first authors further, as well as the even more contentious second to last authorship weightage that may not reflect a senior collaborating author in many cases. Such problems are difficult to solve and arise from the lack of a universal standard where nuances are likely to persist alongside the definition of expertise that is also dependent on the very different needs of different groups. Nevertheless, for the purposes of expert identification within academic settings that is fairer to the last author, the weightage adjustment implemented here may give new upcoming research leads better visibility. Using this platform, we hope to make a small step towards a universal standard and to stimulate further discussion for further improvements. 

The current ACES website is available on webserver.apdskeg.com/aces (last accessed on 28 April 2021) on both desktop and smartphone browsers. A video demonstrating the use of ACES is shown in https://fb.watch/57DYA4KBrj (accessed on 28 April 2021).

## 4. Conclusions

ACES demonstrates the incorporation of NLP for jargon search and a modified last author scoring for the better identification of academia experts from their publications. With modified last author scorings, better credit is given to research leads and provides an added layer of discernment. Altogether, ACES allows for a search of experts based on publicly available lists of publications. With the added feature of deep search, it can be easier for those outside of the specific fields to find experts for business consultancy and collaborators in business, public health, and research.

## Data Availability

ACES belongs to the Agency for Science, Technology, and Research of Singapore, and is for internal use. Interested parties may write to the corresponding author who may make the request to the relevant departments.
